# Androgen Glucuronidation in Mice: When, Where, and How

**DOI:** 10.3390/biology11030403

**Published:** 2022-03-05

**Authors:** Laurent Grosse, Sarah Chouinard, Sophie Pâquet, Mélanie Verreault, Jocelyn Trottier, Alain Bélanger, Olivier Barbier

**Affiliations:** 1 Centre Hospitalié Universitaire de Québec Research Center, Laboratory of Molecular Pharmacology, Endocrinology and Nephrology Axis, Faculty of Pharmacy, Laval University, Québec, QC G1V 0A6, Canada; laurent.grosse@univ-cotedazur.fr (L.G.); sophie.paquet.2@ulaval.ca (S.P.); melanie.verreault@crchudequebec.ulaval.ca (M.V.); jocelyn.trottier@crchudequebec.ulaval.ca (J.T.); 2 Centre Hospitalié Universitaire de Québec Research Center, Faculty of Medicine, Laval University, Québec, QC G1V 0A6, Canada; chouinardsarah@hotmail.com (S.C.); alain.belanger@fmed.ulaval.ca (A.B.)

**Keywords:** androgen hormones, glucuronidation, UDP-glucuronosyltransferase, tissue distribution, kinetic parameters, liquid chromatography coupled with tandem mass spectrometry

## Abstract

**Simple Summary:**

Hormone metabolism can vary from one species to another. In humans, specific UDP-glucuronosyltransferase (UGT) enzymes transform androgens (the male hormones) into glucuronide derivatives, which are easier to eliminate. Whether a similar mechanism also takes place in mice has never been ascertained. This study aimed at addressing this question. Organs and pure Ugt2b enzymes from mice were assayed for their ability to transform several androgens into their glucuronide derivatives. Results show that, as in humans, both murine organs and enzymes are reactive with androgen molecules, and glucuronide derivatives are formed with substrate-, organ- and enzyme-specific manner. In conclusion, these observations revealed that glucuronosyltransferase enzymes from mice works in a similar manner as their human counterparts.

**Abstract:**

Glucuronidation, catalyzed by UDP-glucuronosyltransferase UGT2B enzymes, is a major inactivating and elimination pathway for androgen hormones in humans. Whether Ugt2b enzymes from mice are also reactive with these hormones have never been investigated. The present study aimed at evaluating the capability of murine tissues and Ugt2b enzymes to glucuronidated androgens. The 7 murine Ugt2b (Ugt2b1, 2b5, 2b34, 2b35, 2b36, 2b37 and 2b38) enzymes were cloned and stably expressed into HEK293 cells. In vitro glucuronidation assays were performed with microsomal proteins or homogenates from mice tissues (liver, kidney, intestine, adipose, testis, prostate, epididymis, bulbo, seminal vesicle, mammary glands, uterus, and ovary) and from Ugt2b-HEK293 cells. Male and female livers, as well as male kidneys, are the major sites for androgen glucuronidation in mice. The male liver is highly efficient at glucuronidation of dihydrotestosterone (DHT) and testosterone and is enriched in Ugt2b1 and 2b5 enzymes. Androsterone and 3α-Diol are conjugated in the male kidney through an Ugt2b37-dependent process. Interestingly, castration partially abolished hepatic Ugt2b1 expression and activity, while Ugt2b37 was totally repressed. DHT injection partially corrected these changes. In conclusion, these observations revealed the substrate- and tissue-specific manner in which murine Ugt2b enzymes conjugate androgens. They also evidence how androgens modulate their own glucuronide conjugation in mice.

## 1. Introduction

UDP-glucuronosyltransferase (UGT) enzymes catalyze the glucuronidation reaction, a conjugation process corresponding to the addition of the glucuronic moiety from the UDP-glucuronic acid (UDPGA) co-substrate to various endogenous and exogenous molecules [1]. This reaction converts hydrophobic aglycons into highly polar molecules, and thus facilitates their removal from the human body in bile and urine [2]. Beyond their role in drug (as well as other exogenous compounds) metabolism, UGTs are also essential to the homeostatic equilibrium of numerous endogenous molecules, as varied as steroid and thyroid hormones, bile acids, retinoids and fatty acids [3].

Based on their amino acid sequence homology, UGTs are categorized into two families, namely UGT1 and UGT2 [4]. The UGT1 family comprises 1 subfamily, UGT1A, which is composed of nine members [5]. UGT2 enzymes are further divided into two sub-families, UGT2A and UGT2B [4]. In humans, the three UGT2A enzymes are expressed in the olfactive epithelium and liver and are generally associated to the inactivation of odorant molecules [4]. Seven human UGT2Bs have been identified (UGT2B4, 2B7, 2B10, 2B11, 2B15, 2B17, and 2B28) and are encoded by different genes, all clustered on chromosome 4q13-4q21.1 [4,5]. These enzymes are expressed in numerous tissues, including the liver, as well as steroid target tissues, such as the prostate, breast, skin, testis, and adipose tissue [3]. In mice, seven Ugt2b enzymes (Ugt2b1, 2b5, 2b34, 2b35, 2b36, 2b37, and 2b38) have also been identified [6,7].

C_19_-steroids, such as testosterone, dihydrotestosterone (DHT), androsterone (ADT), and androstane-3α, 17β-Diol (3α-Diol) are preferentially conjugated by UGT2B7, 2B15, and 2B17. Among the numerous endobiotics that are known as substrates for UGT2Bs, androgens received particular attention during the last two decades [3,8,9,10,11,12,13,14,15]. This intense research effort first established the quantitative importance of androgen glucuronides in the human circulation where ADT-glucuronide (ADT-G) and 3α-Diol-G are the most abundant androgen forms [16,17], and then identified the UGT2B7, 2B15 and 2B17 isoforms as highly reactive in front of those androgens [3]. Indeed, UGT2B7 catalyzes glucuronide conjugation of the 3-hydroxyl position of androsterone and 3α-Diol [18,19], whereas the UGT2B15 isoform is stereoselective for the glucuronidation of the 17β-hydroxyl position of testosterone, DHT and 3α-Diol [3]. By contrast, UGT2B17 is able to use both the 3-hydroxyl position of ADT and 17β-hydroxyl position of 3α-Diol as an acceptor group for the glucuronosyl moiety [3].

While numerous evidences have pointed out glucuronidation and UGT enzymes as major controllers of androgen action and metabolism [10,15], mechanistic investigations are limited to cell cultures, since no appropriate animal models have been identified until now. Recent investigation demonstrated that, as humans, mice express Ugt2b enzymes (Ugt2b1, 2b5, and 2b34–2b38) [6,7], suggesting that these animals possess a similar enzymatic equipment for androgen glucuronidation. The current study was therefore designed to analyze the ability of murine tissues to glucuronide androgens, and to decipher the contribution of each of the 7 Ugt2bs from mice in this metabolic reaction.

## 2. Experimental Procedures

### 2.1. Materials

UDP-glucuronic acid and all aglycons were obtained from Sigma (St. Louis, MO, USA) and ICN Pharmaceuticals, Inc. (Québec, QC, Canada). Lipofectin, Zeocin, and expression vectors were purchased from Invitrogen (Burlington, ON, Canada). Proteins assay reagents were obtained from Bio-Rad Laboratories, Inc. (Richmond, CA, USA). The Pfu Turbo DNA polymerase was obtained from Stratagene (La Jolla, CA, USA). Restriction enzymes and other molecular biology reagents were purchased from Roche Molecular Biochemicals (Indianapolis, IN, USA). Human embryonic kidney 293 cells (HEK293) were obtained from the American Type Culture Collection (Rockville, MD, USA). Ammonium formate was from Aldrich Chemical (Milwaukee, WI, USA), and high-performance liquid chromatography (HPLC)-grade methanol was provided by VWR Canlab (Montréal, QC, Canada).

### 2.2. Animal Experiments and Tissue Collection

Animal studies were performed in compliance with the Guidelines for Care and Use of Experimental Animals from the Canadian Council for Laboratory Animal Care. All experiments were approved by the Animal Care Council of the CHU de Québec Research Center (approbation number no. 12-078-1). Animals were housed in a 12 h daylight environment (12 h daylight cycle, lights off at 18:00 h) with food and water ad-libitum in the animal facility of the CHU de Québec Research Center. For gonadectomy studies, twelve 90-day old C57BL/6N male mice were castrated and received buprenorphine (Sigma Aldrich, Oakville, ON, Canada; 0.1 mg/kg) on day 0 (surgery). Six male mice were sham-operated and were used as control. One day after surgery, and every following days for two weeks, six gonadectomized mice were injected subcutaneously with DHT (0.1 mg/day), while the remaining castrated animals, as well as the sham-operated ones, were injected with saline. At the end of the two-week experiment, isoflurane (Baxter Corporation, Mississauga, ON, Canada) was used to anesthetize the animals at sacrifice. Blood was collected through cardiac punctures and was added to 100 µL EDTA (100 mM). Plasma was isolated by centrifugation at 3000 rpm for 5 min. Tissues were collected and freed from fat connective tissues immediately after death. Tissues were washed in KCl 1.15% and quickly frozen on dry iced. Tissues were kept at −80 °C for subsequent RNA and protein isolations.

### 2.3. RNA Isolation and Quantitative RT-PCR

Total RNA was isolated from mice tissues (20 mg) according to the TRI Reagent acid: phenol protocol as recommended by the supplier (Molecular Research Center Inc., Cincinnati, OH, USA). One µg of total RNA was reverse-transcribed using random hexamer primers (150 ng) and 200 units of Superscript II reverse-transcriptase according to manufacturer’s instructions (Invitrogen, Burlington, ON, Canada).

Transcript levels of the seven Ugt2b were quantified through real time PCR experiments, using isoform-specific primers (Table 1), and a real-time PCR ABI Prism 7500 instrument from Applied Biosystems (Foster City, CA, USA). For each reaction, the final volume of 20 µL was composed of 10 µL of SYBR Green PCR mix, 2 µL of each primer, and 6 µL of a RT product diluted 1/50. Copy numbers were obtained through linear regression with a standard curve constructed with five log concentrations of each UGT2b cDNA. Integrity of the reverse transcription reaction was assessed by analyzing mRNA levels of the house keeping gene PPIA. Specificity of amplification for each Ugt2b was ensured by direct sequencing of PCR products.

### 2.4. Murine Ugt2b cDNA Cloning and Stable Expression in HEK293 Cells

UGT2b constructs were obtained through PCR amplification using isoform-specific primers designed to generate PCR products comprising the previously reported open reading frames. NheI and XhoI restriction sites were added to the 5′-untranslated extremity of forward and reverse primers, respectively (Table 2). cDNA amplification was performed using Pfu Turbo Polymerase enzyme (Stratagene; Bellingham, WA, USA) and a GeneAmp PCR 9700 system (ThermoFisher Scientific; Waltham, MA, USA) in the presence of 2 µL of RT products synthesized from the male mouse liver. PCR products were subsequently purified using the QIAquick^®^ PCR Purification Kit (Qiagen, Mississauga, ON, Canada) and inserted into the pCR^®^II-TOPO^®^ (ThermoFisher Scientific; Waltham, MA, USA) vector, as recommended by the supplier. These Ugt2b cDNA were sub-cloned into the NheI/XhoI digested pcDNA4 vector (ThermoFisher Scientific; Waltham, MA, USA) with the Rapid DNA Ligation Kit (Roche; Mississauga, ON, Canada). Thereafter, plasmids encoding Ugt2b1, 2b5, 2b34, 2b35, 2b36, 2b37, and 2b38 (2 µg) were transfected into HEK293 cells using the Lipofectamine reagent (ThermoFisher Scientific; Waltham, MA, USA) as recommended by the supplier. Stable Ugt2b-expressing clones were selected for 1 month with Zeocin (32.5 μg/mL; ThermoFisher Scientific; Waltham, MA, USA).

### 2.5. Microsome Isolation, Western-Blotting, and Glucuronidation Assays

Microsomes from human liver (Lot # H0610) were from XenoTech (Kansas city, MO, USA). Tissues or Ugt2b-overexpressing HEK293 cells were homogenized with a polytron (Brinkmann Instruments, Inc., Westbury, NY, USA). Microsome pellets were extracted by differential centrifugation as previously described [20] and resuspended at a 5 µg/µL concentration.

For western-blotting, microsomal proteins (5 µg) or cell/tissue homogenates (25–50 µg) were size-separated on a 10% SDS-polyacrylamide gel. Gels were transferred onto nitrocellulose membranes and probed with the anti-UGT2B (EL-93, dilution 1/2000), anti-actin (1/5000) and anti-calnexin (1/2000) antibodies as previously reported [21,22]. The homemade EL-93 (anti-UGT2B) was obtained as previously described [20]. An anti-rabbit IgG antibody coupled with peroxidase (dilution 1/10,000) (Amersham Pharmacia Biotech, Oakville, ON, Canada) was used as second antibody, and the resulting immunocomplexes were detected with ECL (ThermoFisher Scientific; Waltham, MA, USA) and exposed on a Hyperfilm^TM^ for 15 s (Kodak Corp., Rochester, NY, USA).

Glucuronidation assays were performed in the presence of 40–50 µg homogenates using the previously reported glucuronidation assay buffer [20]. All screening assays were performed in the presence of 100 µM substrates (testosterone, DHT, ADT, 3α-Diol or the positive control 4-methylumbelliferone) at 37 °C for 1 h in a final volume of 100 µL. Assays were ended by adding 100 µL of methanol with 0.02% butylated hydroxytoluene. Kinetic experiments were performed with the presence of increasing substrate concentrations (1–200 µM).

### 2.6. Steroid Quantification

The formation of glucuronide conjugates (DHT-[17]G, ADT-[3]G, 3α-Diol-[3 or 17]G and 4-MU-G) was analyzed by liquid chromatography coupled with tandem mass spectrometry (LC/ESI–MS/MS) as already reported [19,23].

### 2.7. Statistical Analysis

All data are presented as mean ± standard deviation (S.D.). The statistical significance of differences was determined through the Student *t* test using the JMP V7.0.1 program (SAS Institute, Inc.; Cary, NC, USA).

## 3. Results

### 3.1. Gender- and Tissue-Specific Androgen Glucuronidation and Ugt2b Expression in Mice

**Figure 1:**
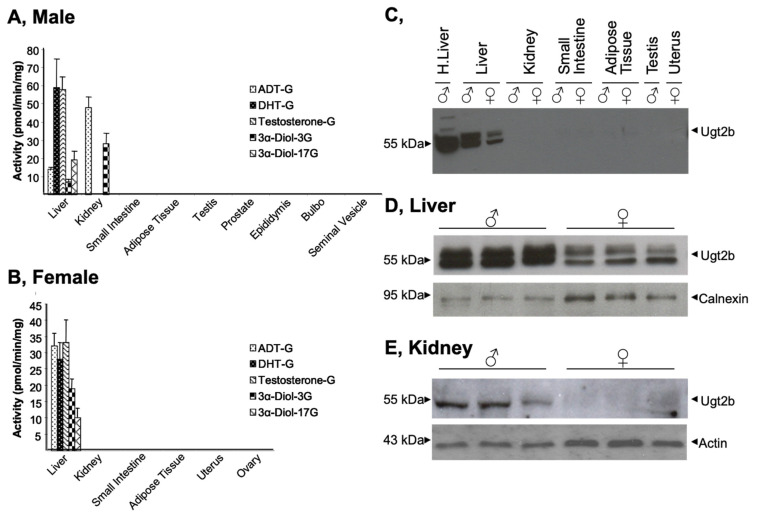
Tissue- and gender-specific distribution of Ugt2b expression and androgen-conjugating activities in mice. (**A**,**B**) Glucuronidation assays were performed with homogenates (40 µg) of male (**A**) and female (**B**) murine tissues in the presence of 100 μM of androgens for 1 h. Assays were performed in triplicates with tissues from three animals. The formation of androgen glucuronides was quantified by LC–MS/MS analyses. (**C**–**E**) Tissue homogenates (40 μg, (**C**)) or microsomal proteins (5 µg; (**D**,**E**)) were size-separated on 10% SDS-PAGE, transferred onto nitrocellulose membranes and hybridized with an anti-UGT2B (EL-93, 1/2000) antibody. (**D**,**E**) Membranes were subsequently immunoblotted with an anti-calnexin ((**D**) 1/2000) or an anti-actin ((**E**) 1/5000) antibody to ensure an equal loading of proteins in each lane. ADT: androsterone; DHT: dihydrotestosterone; 3α-Diol: androstane-3α, 17β-diol. Full western blot are provided as supplementary Figure S1.

The capability of murine tissues (namely the liver, kidney, intestine, adipose tissue, testis, prostate, epididymis, bulbo gland, and seminal vesicles) to convert androgens into glucuronides was evaluated using in vitro glucuronidation assays performed in the presence of 100 µM of testosterone, DHT, ADT or 3α-Diol (Figure 1A,B). These tissues were selected based on their reported ability to glucuronidated androgen hormones in humans [3,24]. The formation of androgen glucuronide was detected only with homogenates from liver (male and female) and kidney (male), while all other tissues were unable to glucuronidate any of the four hormones assayed (Figure 1A,B). In the same vein, while male kidney extracts were highly reactive to generate ADT-G and 3α-Diol-3G (Figure 1A), those from female animals were unreactive toward those substrates (Figure 1B). Interestingly, the male liver was highly reactive for steroids, such as testosterone and DHT, while the formation of ADT-G, 3α-Diol-3, and -17G only occurred at a three-fold lower rate (Figure 1A). Similarly, experiments performed with female livers lead to lower formation of 3α-Diol-glucuronide conjugates (3α-Diol-3 and -17G) than other glucuronides (Figure 1B). Moreover, DHT and testosterone were glucuronidated twice more in male than female samples, while the hepatic formation of ADT-G (3-fold) and 3α-Diol-3 and -17G (2-fold) sustained an inverse sex-related variation. Taken together, these observations indicate that, in mice, androgen glucuronidation might be restricted to the liver and kidney for males and only to the liver for female.

**Figure 2:**
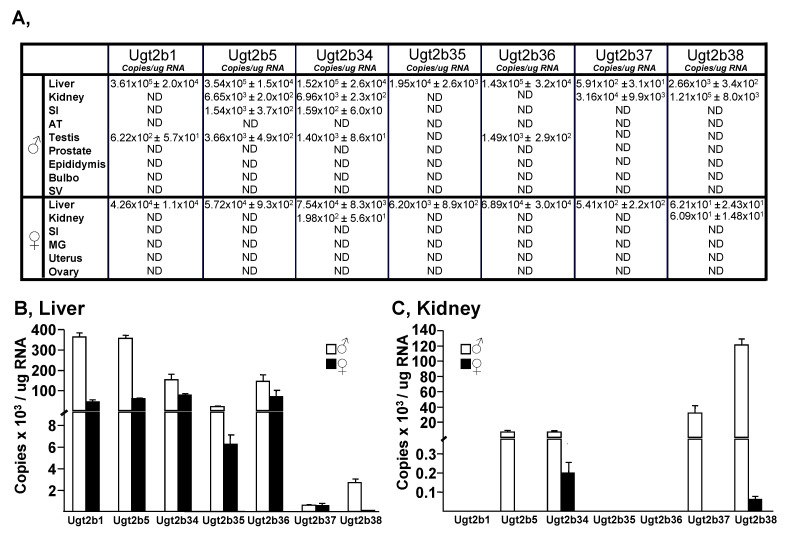
Tissue distribution of murine Ugt2b transcripts. Total RNA from male and female mice (n = 3/sex) tissues were extracted, reverse-transcribed, and analyzed for the expression of the seven Ugt2b enzymes through qRT-PCR analyses (Table 1). The Ugt2b copy numbers in all tissues (**A**) including Liver (**B**) and Kidney (**C**) were obtained using linear regression with a standard curve that included five log concentrations of the Ugt2b plasmids. Experiments were performed in triplicate. SI, small intestine; AT, adipose tissue; SV, seminal vesicle; MG, mammary gland. ND: not detected.

Subsequent western blot analyses confirmed that Ugt2b proteins are detected in homogenates from male and female livers, but only from male kidneys (Figure 1C–E). Moreover, Ugt2b proteins are more abundant in livers from male homogenates than in female ones (Figure 1C). To further confirm the tissue- and gender-specific manner in which murine Ugt2b enzymes are expressed, the same tissues were analyzed for Ugt2b mRNA levels using isoform-specific qRT-PCR determination (Figure 2). As indicated in Figure 2A, none of the transcripts was detected in peripheral tissues such as the adipose tissue, prostate, epididymis, bulbo, seminal vesicle, mammary gland, uterus and ovary (Figure 2A). Ugt2b1, Ugt2b5, Ugt2b34, and Ugt2b36 transcripts were detected in testis but only at a far lower level than in the liver (Figure 2A). In this last tissue, all transcripts were quantified in both male and female samples (Figure 2A), but with a certain level of variability (Figure 2B). Indeed, while female livers contained similar concentrations of Ugt2b1, Ugt2b5, Ugt2b34, and Ugt2b36 messengers, the male RNAs were particularly enriched in Ugt2b1 and Ugt2b5 transcripts. Nevertheless, the expression of Ugt2b37 and Ugt2b38 was low in liver from both sexes (Figure 2B). Interestingly, these two isoforms were the most abundantly detected in male kidneys, while being absent (Ugt2b37) or barely detected (Ugt2b38) in female kidney RNAs (Figure 2C). Finally, Ugt2b5 and Ugt2b34 mRNAs were also present in renal RNA samples, and in far higher concentrations in the male compared to female samples (Figure 2C).

Overall these observations reveal that in vitro glucuronidation occurs only in murine liver (male and female) and kidney (male) from mice, and accordingly with previous reports [6,7], reinforce the tissue- and gender-specific manner in which Ugt2b enzymes are expressed in this species. These data also suggest that selected Ugt2b enzymes may be responsible for the tissue-selective glucuronidation of androgens in the murine liver and kidney.

### 3.2. Androgen Selectivity of Murine Ugt2b Enzymes

**Figure 3:**
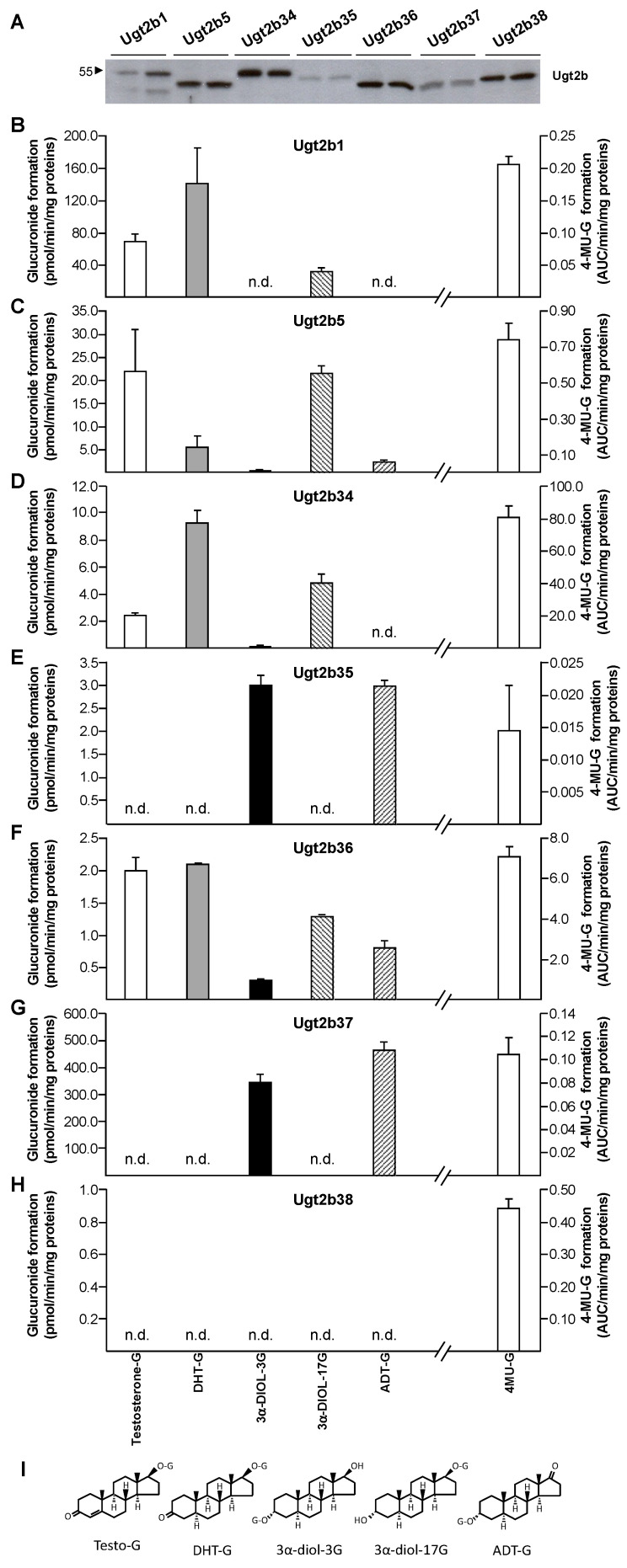
Stereoselectivity of male liver, kidney and recombinant murine Ugt2b enzymes for androgen glucuronidation. (**A**) Homogenates from Ugt2b-HEK293 cells (25 µg) were size separated on 10% SDS-PAGE, transferred onto nitrocellulose membranes and hybridized with the anti-UGT2B (EL-93; 1/2000). (**B**–**H**) Homogenates (50 µg) from Ugt2b1- (**B**), 2b5- (**C**), 2b34- (**D**), 2b35- (**E**), 2b36- (**F**), 2b37- (**G**), and 2b38-HEK293 (**H**) cells were incubated in a glucuronidation assay buffer for 1 h in the presence of 100 µM of androsterone (ADT), dihydrotestosterone (DHT), testosterone, androstane-3α, 17β-diol (3α-Diol), or 4-methylumbelliferone (4-MU, positive control). Glucuronidated products were quantified by LC–MS/MS analyses. (**I**) Numbered structures are illustrated. Data represent the mean of two experiments performed in triplicate. Nd: not detected. Full western blot are provided in supplementary Figure S2.

To evaluate the Ugt2b-dependent specificity of androgen glucuronidation in mice, we next cloned, stably expressed in human HEK293 cells, and evaluated the reactivity of each of the seven murine Ugt2b enzymes with androgen substrates (testosterone, DHT, ADT, and 3α-Diol) and 4-methylumbelliferone (4-MU) (Figure 3). The presence of each enzyme in the corresponding Ugt2b-HEK293 cell line was assessed through western-blotting (Figure 3A). As expected, Ugt2b proteins showed different sizes reflecting variables sizes (from 529 to 532 amino acids), and the *N*-glycosylation status as previously observed with other UGT enzymes [25] and all cell homogenates were reactive with the positive control substrate 4-MU (Figure 3B–H). The conversion of 3α-Diol and ADT into their respective 3α-Diol-3G and ADT-G derivatives was catalyzed at high levels only in the presence of homogenate from Ugt2b37-HEK293 cells (Figure 3G). While exhibiting the same substrate selectivity, Ugt2b35 only formed 3α-Diol-3G and ADT-G at low levels (Figure 3E). Ugt2b1 was the most active enzyme for the production of testosterone-G, DHT-G and 3α-Diol-17G (Figure 3B), while Ugt2b5 and Ugt2b34 were also able to generate these glucuronide derivatives but at lower levels (Figure 3C,D). Interestingly, all glucuronide derivatives were detected-but at low levels-when their unconjugated substrates were incubated in the presence of Ugt2b36-HEK293 cell homogenates (Figure 3F). By contrast, Ugt2b38 was inefficient at converting any of the androgens assayed into their glucuronide conjugates (Figure 3H).

Overall, these experiments point-out: (i) the Ugt2b1, 2b5, and 2b34 enzymes as the potential contributors of the elevated rates of testosterone-G, DHT-G, and 3α-Diol-17G formation in the presence of liver extracts (Figure 1A,B); and (ii) the Ugt2b37 enzyme as the probable catalyzer of ADT-G and 3α-Diol-3G formation in male kidney (Figure 1A). To investigate such a possibility, we determined and compared the kinetic parameters of androgen glucuronidation by microsomes from male liver and kidney samples or Ugt2b-HEK293 cells (Table 3). When assayed for testosterone glucuronidation, liver and Ugt2b1 extracts exhibited similar affinity (K_M_) values of 29.2 ± 1.7 µM and 20.3 ± 0.7 µM, respectively. By contrast, the affinity of the Ugt2b5 enzyme (65.5 ± 8.6 µM) was two-times lower when compared to the liver. Similarly, Ugt2b1 also had the closest K_M_ values for DHT when compared to the liver (17.8 ± 0.7 and 24.1 ± 1.0 µM, respectively), while Ugt2b5 (35.6 ± 11.5 µM) and Ugt2b34 (54.9 ± 2.9 µM) exhibited lower affinities (Table 3). Interestingly, microsomal proteins purified from the murine liver (K_M_ = 19.3 ± 2.0 µM) and from HEK293 expressing Ugt2b1 (K_M_ = 17.8 ± 2.0 µM), Ugt2b5 (K_M_ = 22.5 ± 5.1 µM) or Ugt2b34 (K_M_ = 16.3 ± 4.8 µM) exhibited all similar values for the conversion of 3α-Diol into 3α-Diol-17G (Table 3). Finally, kidney and Ugt2b37-HEK293 cells also exhibited remarkable similar K_M_ values for 3α-Diol-3G and ADT-G conjugations (Table 3).

Overall, these last observations identify Ugt2b1 and Ugt2b37 as the most active androgen glucuronidating enzymes in the murine liver and kidney, respectively.

### 3.3. Androgens Control Their Own Glucuronidation in the Murine Liver and Kidney

**Figure 4:**
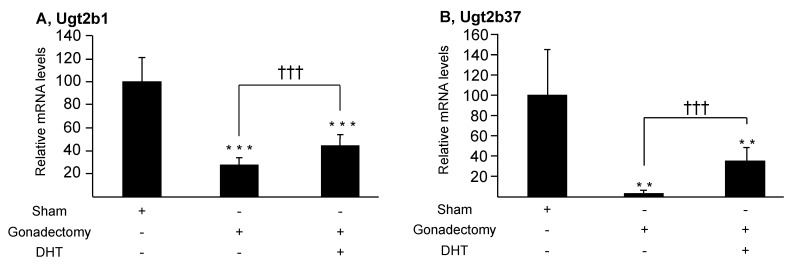
Gonadectomy and/or DHT differentially affect mRNA expression of the androgen-conjugating Ugt2b1 and Ugt2b37 enzymes in male liver and kidney, respectively. Male mice (90 days old, n = 6/group) were either sham-operated (Sham), gonadectomized, and/or treated with 0.1 mg DHT/day for 14 days, before euthanasia. Livers and kidneys were collected and subsequently investigated for Ugt2b1 (**A**) or Ugt2b37 (**B**) mRNA levels through qRT-PCR analyses. Ugt2b transcript copy numbers were obtained using linear regression with a standard curve that included five log concentrations of the corresponding Ugt2b plasmid and expressed as a percentage of copy numbers of control (sham) animals. Statistical analyses: Student’s T test. **: *p* < 0.005, ***: *p* < 0.001: versus sham; and ^†††^: *p* < 0.001 gonadectomized versus DHT-treated gonadectomized animals.

**Figure 5:**
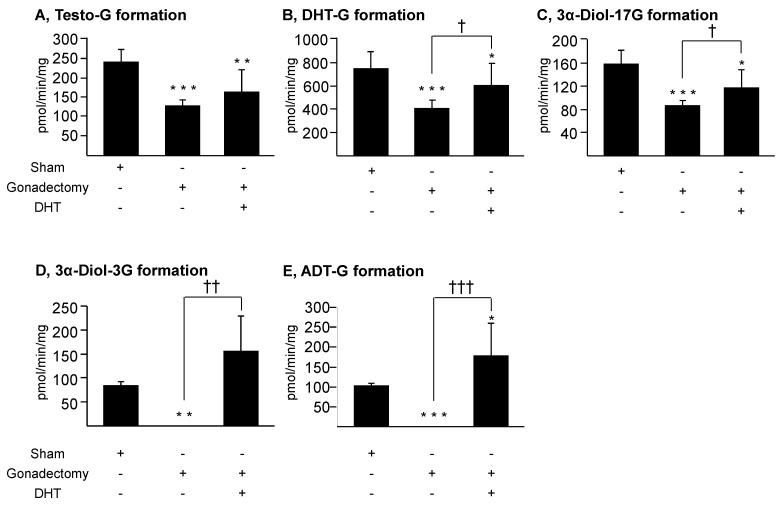
Gonadectomy and/or DHT differentially affect androgen glucuronidation in the male liver and kidney. Male mice (90 days old, n = 6/group) were either sham-operated (sham), gonadectomized and/or treated with 0.1 mg/day DHT for 14 days, before euthanasia. Livers and kidneys were collected, homogenized, and assayed for androgen glucuronidation as described in the materials and methods section. Glucuronidation assays were performed with liver (**A**–**C**) or kidney (**D**,**E**) homogenates in the presence of 100 μM androgens for 1 h. The formation of androgen glucuronides was quantified by LC–MS/MS analyses. Statistical analyses: Student *t* test: *: *p* < 0.05, **: *p* < 0.005, ***: *p* < 0.001: versus sham; and ^†^: *p* < 0.05, ^††^: *p* < 0.005, ^†††^: *p* < 0.001 gonadectomized versus DHT-treated gonadectomized. ADT: androsterone; DHT: dihydrotestosterone; 3α-Diol: androstane-3α, 17β-diol; G: glucuronide.

To evaluate whether androgens control their own glucuronidation in mice, we next investigated whether DHT also affects the expression and activity of the Ugt2b1 and Ugt2b37 enzyme in male mice liver and kidney. For this purpose, 90-day old animals were sham-operated or gonadectomized and/or injected with DHT (0.1 mg) (Figure 4 and Figure 5). Accordingly to previous reports [7], castration resulted in a strong reduction of hepatic Ugt2b1 mRNA levels in liver extracts, while the renal mRNA content in Ugt2b37 transcripts was also reduced (Figure 4). In DHT-treated gonadectomized animals, hepatic Ugt2b1 and renal Ugt2b37 mRNA expression were only partially restored. In addition, glucuronidation assays reveal that gonadectomy caused a significant reduction of the formation of testosterone-G, DHT-G, and 3α-Diol-17G by liver enzymes, and of 3α-Diol-3G and ADT-G by renal proteins from homogenates (Figure 5). While DHT injections fully restored androgen glucuronidation in the kidney (Figure 5D,E), the hepatic formation of androgen glucuronides was higher than after castration but remained significantly reduced in sham-operated samples (Figure 5A–C).

Overall, these observations demonstrate that gonadal androgens have the potential of controlling their own hepatic and renal glucuronidation in mice.

## 4. Discussion

The present study provides the first comprehensive analysis of the expression and activity of murine Ugt2bs in the control of androgen conjugation capabilities in mice. In vitro assays identified the liver as the main site for active androgen conjugation. These assays also revealed that kidney tissue is efficient at conjugating 5α-reduced hormones, namely ADT and 3α-Diol. We also evidenced that this stereo-selectivity for androgen glucuronidation reflects the differential expression of three enzymes: Ugt2b1 and Ugt2b5, which are expressed at high level in the liver and Ugt2b37 in the kidney.

The principal objective of the present study was to evaluate the contribution murine tissues and Ugt2bs in androgen glucuronidation. Our results clearly establish that, as human tissues and UGT2B enzymes [26], the murine liver and kidney, as well as the Ugt2b1 (liver) and Ugt2b37 (kidney) enzymes are highly reactive with these substrates, in vitro. In contrast to humans where ADT-G and 3α-Diol-G are the most abundant circulating androgen forms [16,17], the murine plasma does not contain any detectable androgen glucuronide [16,17]. However, the present study establishes that this absence of circulating androgen glucuronide in mice cannot be attributed to the inability of the Ugt2b enzymatic machinery to conjugate androgens. Thus, such an absence may actually reflect other physiological differences between rodents and higher mammals. Accordingly, humans and mice also display major dissimilarities in terms of androgen hormone synthesis [24]. For example, in humans, both the gonads (with testosterone) and the adrenals (with DHEA and DHEA-S) contribute to the production of androgenic precursors, while in rodents, the unique source of active DHT comes from the gonadal secretion of testosterone (Reviewed in [24]). Together, these data show that the androgen physiology differs between rodents and humans, both in terms of hormone synthesis and metabolism, and results of the current study reveals that these differences are not linked to the glucuronidation reaction itself. Indeed, not only murine Ugt2b enzymes are able to use androgens as substrates, but their enzymatic properties are also close to those reported for human enzymes [3,20,22,25]. For example, the K_M_ values for androgen glucuronidation by the human UGT2B7, UGT2B15 and UGT2B17 are in a micromolar range [3,20,22,25] which resembles to those of the murine Ugt2b enzymes, as demonstrated in the present study. Even in terms of substrate stereo-specificity, the murine enzymes resemble to human ones. For example, both murine Ugt2b1 and human UGT2B15 are highly selective glucuronidating enzymes for the 17β-hydroxyl position of testosterone, DHT, and 3α-Diol [3,22], while the high affinity of Ugt2b37 for using the 3α-position of ADT and 3α-Diol is similar to what is reported for the human UGT2B7 isoform, an enzyme highly expressed in the human kidney [3,18]. To summarize, the results presented here demonstrate that Ugt2b enzymes exhibit similar capabilities and properties for androgen glucuronidation as human isoforms.

Despite their enzymatic similarities, murine androgen-conjugating Ugt2b enzymes display a very restricted tissue-distribution of their expression when compared to human enzymes. Indeed, accordingly with previous investigations [6], our investigations revealed that murine Ugt2b enzymes are almost exclusively expressed in the liver and kidney and are absent from major androgen target tissues such as the prostate, adipose tissues or epididymis. Furthermore, even when assayed through in vitro glucuronidation experiments, these targets appears as androgen glucuronidating-deficient tissues. Numerous reports have previously demonstrated that even if the liver and kidney are important sites for androgen glucuronidation in humans, this conjugation reaction also takes place in several peripheral tissues, including those listed above (reviewed in [24]). Not only androgen glucuronidation takes place in human peripheral organs, but recent discoveries demonstrate that in human androgen target tissues, such as the prostate, glucuronidation serves as a termination mechanism for the androgen signaling [26,27]. In this context, the present demonstration that peripheral tissues are unable to glucuronidate androgens in mice highlight another major difference between the two species, as of controlling androgen signaling.

Furthermore, the present study also demonstrates that the removal of the endogenous site of androgen production (i.e., gonadectomy) causes a drastic reduction in Ugt2b1 (liver) and Ugt2b37 (kidney) expression, resulting in a significant decrease of androgen glucuronidation capabilities in both tissues. Nevertheless, providing exogenous hormones (namely DHT) restored, at least partially, these parameters. Taken together, these observations suggest that androgens serve as positive regulators for Ugt2b1 and Ugt2b37 expression, and by so, are able to stimulate their own glucuronidation. Again, this situation differs from what has been reported in humans, where androgens and their nuclear receptor were identified has negative regulators of the expression of the androgen-conjugating UGT2B15 and UGT2B17 enzymes [26,27]. These observations therefore denote another major difference between human and murine Ugt2b enzymes, i.e., their differential response to androgen in terms of expression and activity.

## 5. Conclusions

In conclusion, the present investigations revealed the substrate- and tissue-specific manners in which murine Ugt2b enzymes conjugate androgens. They also evidence how androgens modulate their own glucuronide conjugation in mice and provide the first evidence of a species-specific manner in which androgens control UGT2B expression.

## Figures and Tables

**Table 1:** Primer sequences for real time PCR analyses of mouse Ugt2b.

Real-Time Pcr Primers			
	Forward	Reverse	Annealing Temperature
PPIA	5′-TCCTGGCATCTTGTCCATG-3′	5′-CATCCAGCCATTCAGTCTTG-3′	54 °C
Ugt2b1	5′-TATGTTGCAGGTGTTGCT-3′	5′-GTCCCAGAAGGTTCGAAC-3′	60 °C
Ugt2b5	5′-GGGACTCATTTTACAGTGAG-3′	5′-CATGTTACTAACCATTGACC-3′	56 °C
Ugt2b34	5′-AGCCCCTGCCTAAGGAAATA-3′	5′-GAGTGTTGGAGCCCAATGTC-3′	60 °C
Ugt2b35	5′CCAGACATTTACAGAGAAGG-3′	5′CTGTCATGTTACTGACCATC-3′	60 °C
Ugt2b36	5′-TTGTTCAGAGCTCTGGAGAG-3′	5′-GATGACCAAGAAGATCATTT-3′	56 °C
Ugt2b37	5′-ATTTGGAGTTTCCTCACCCGA-3′	5′-TAGATTGCCTCATAGACACTG-3′	60 °C
Ugt2b38	5′-GCAACTTTAGGACACAATACG-3′	5′-ACTTCCTCCAGTGCATTGAGT-3′	60 °C

**Table 2:** Primer used for Ugt2b cDNA cloning (restriction sites are underlined).

Primer	Sequence	RefSeq	Full Length cDNA
Ugt2b1 NheI Forward	5′-CTAGGCTAGCATCTAGTCAGTGATGTGGTTAGAAG	AC119816.5	2560 bp
Ugt2b1 XhoI Reverse	5′-TCGACTCGAGCAGGACTCTCTGCTTCAGCCTTCAT		
Ugt2b5 NheI Forward	5′-CTAGGCTAGCAGCAAATGGACTGTGAGAGAAGGAT	NM_009467.3	1901 bp
Ugt2b5 XhoI Reverse	5′-TCGACTCGAGAGGCTGAAAGTTTGTTCATGTAGTT		
Ugt2b34 NheI Forward	5′-CTAGGCTAGCGCCTGAAGTTAACCAA	NM_153598.2	3048 bp
Ugt2b34 XhoI Reverse	5′-TCGACTCGAGTGAAGGACCCTAAATCATTGCCTCC		
Ugt2b35 NheI Forward	5′-CTAGGCTAGCGTTAACAGAAGCCCTTTGAC	NM_172881.3	3358 bp
Ugt2b35 XhoI Reverse	5′-TCGACTCGAGTTCTTCCTTTTCTTTGCC		
Ugt2b36 NheI Forward	5′-CTAGGCTAGCACTCTGAAGAGAAGAACA	NM_001029867.1	1888 bp
Ugt2b36 XhoI Reverse	5′-TCGACTCGAGATGCATTATCAATGAGT		
Ugt2b37 NheI Forward	5′-CTAGGCTAGCGCAAATGAACTGTGAAGAGAAGGAT	AC100269.7	1879 bp
Ugt2b37 XhoI Reverse	5′-TCGACTCGAGAAATAGATGGGATTTTTGAAAATGC		
Ugt2b38 NheI Forward	5′-CTAGGCTAGCCCCACGCGTCCGGGATT	NM_133894.2	1894 bp
Ugt2b38 XhoI Reverse	5′-TCGACTCGAGATGGATCAGTATCCACAGATTTAC		

**Table 3:** Kinetic parameters of androgens glucuronidation by microsomal proteins from male liver, kidney, and recombinant Ugt2b proteins.

Androgen-Glucuronide	Tissue/Enzyme	Vmax_app_ (pmol/min/mg Proteins)	K_M_ (µM)
Testosterone-G	Liver	1611.3 ± 56.1	29.2 ± 1.7
	Ugt2b1	51.2 ± 1.5	20.3 ± 0.7
	Ugt2b5	186.5 ± 1.0	65.5 ± 8.6
DHT-G	Liver	3987.0 ± 95.5	24.1 ± 1.0
	Ugt2b1	238.5 ± 0.3	17.8 ± 0.7
	Ugt2b5	50.5 ± 2.1	35.6 ± 11.5
	Ugt2b34	43.1 ± 1.7	54.9 ± 2.9
3α-Diol-17G	Liver	485.3 ± 24.5	19.3 ± 2.0
	Ugt2b1	34.8 ± 1.7	11.7 ± 2.0
	Ugt2b5	115.2 ± 12.4	22.7 ± 5.1
	Ugt2b34	8.5 ± 0.6	16.3 ± 4.8
3α-Diol-3G	Kidney	215.9 ± 2.9	2.0 ± 0.1
	Ugt2b37	1774.1 ± 51.6	3.9 ± 1.5
ADT-G	Kidney	262.8 ± 2.2	2.3 ± 0.8
	Ugt2b37	2091.0 ± 50.8	3.6 ± 0.4

Results are expressed as mean ± S.D. of 2 experiments performed in triplicate. K_M_: Michaelis constant; Vmax_app_: apparent maximal velocity; ADT: androsterone; DHT: dihydrotestosterone; 3α-Diol: androstane-3α, 17β-diol.

## Data Availability

All data could be obtained upon request to the authors.

## Supplementary Materials

The following supporting information can be downloaded at: https://www.mdpi.com/article/10.3390/biology11030403/s1, Figure S1: Full western blots of Figure 1; Figure S2: Full western blots of Figure 3.

## Abbreviations

ADT, androsterone, ADT-G, androsterone-glucuronide; AT, adipose tissue; 3α-Diol, androstane-3α, 17β-Diol; DHT, dihydrotestosterone; HEK293, human embryonic kidney 293 cells; LC/ESI–MS/MS, liquid chromatography coupled with tandem mass spectrometry; 4-MU, 4-methylumbelliferone; MG, mammary gland; SI, small intestine; SV, seminal vesicle; UDPGA, UDP-glucuronic acid; UGT, UDP-glucuronosyltransferase
