# Supplementary material for: Androgen Glucuronidation in Mice: When, Where, and How
Source: Biology (Basel). 2022 Mar 5;11(3):403. doi: 10.3390/biology11030403 (PMC8945853; doi:10.3390/biology11030403)

Androgen glucuronidation in mice: when, where and how.

Laurent Grosse [1], Sarah Chouinard [2], Sophie Pâquet [1], Mélanie Verreault [1], Jocelyn Trottier [1], Alain Bélanger [2] and Olivier Barbier [1,*]

SUPPLEMENTARY MATERIAL : SUPPLEMENTARY FIGURES S1 AND S2: Full Western blots of Figures 1 and 3

SUPPLEMENTARY FIGURE S1: Full western blots of Figure 1

correspond to figure 1, panel C

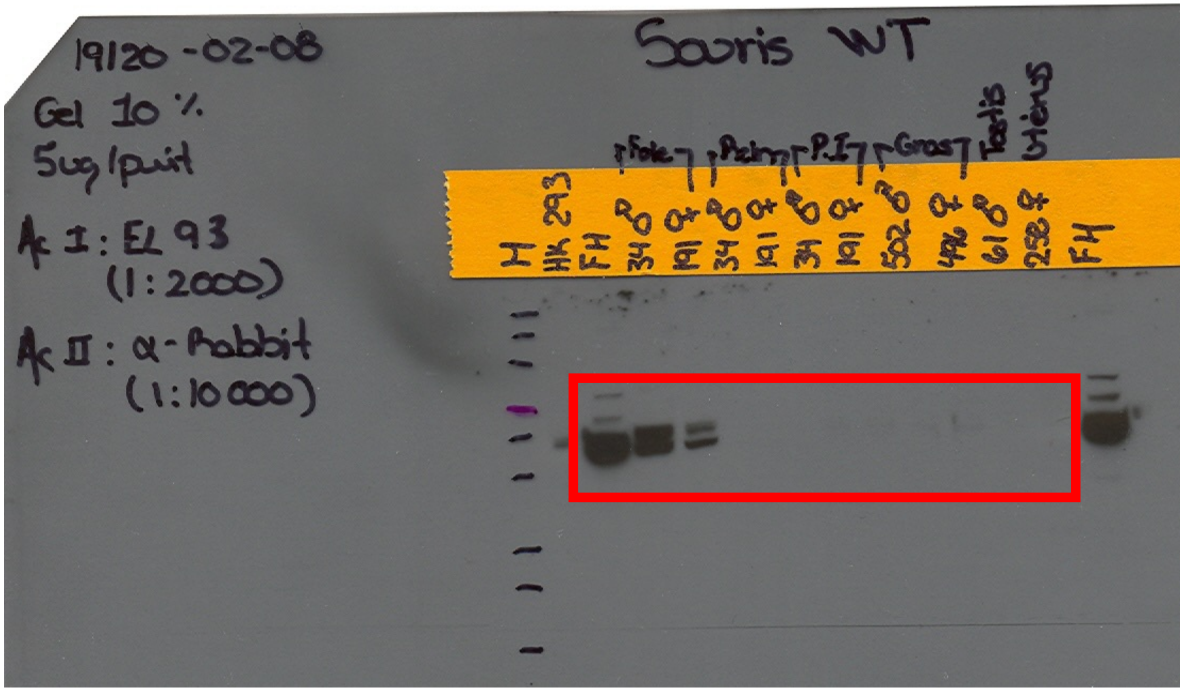

SUPPLEMENTARY FIGURE S1: Full western blots of Figure 1

correspond to figure 1, panel D – top panel

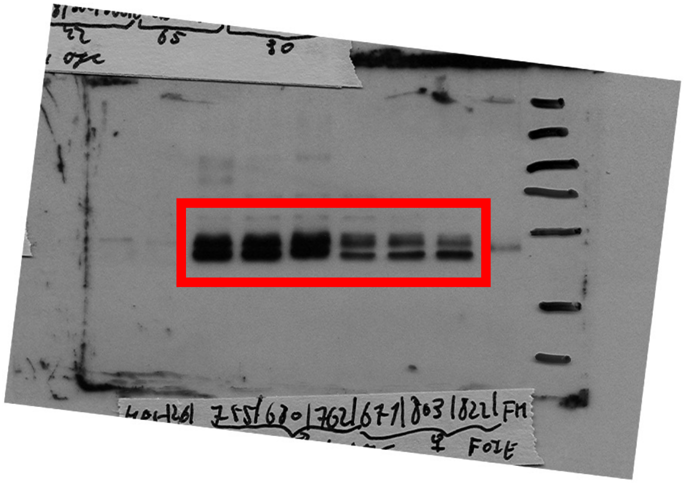

SUPPLEMENTARY FIGURE S1: Full western blots of Figure 1

correspond to figure 1, panel D – lower panel

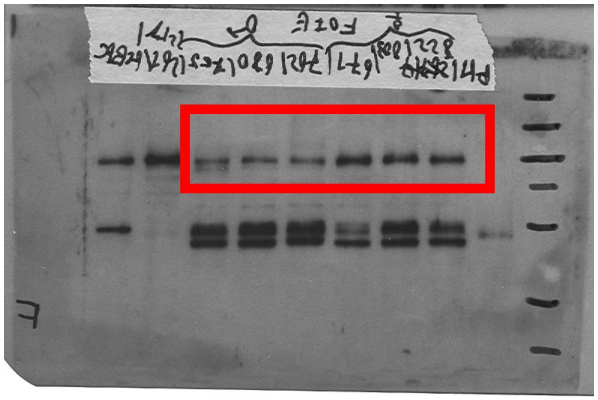

SUPPLEMENTARY FIGURE S1: Full western blots of Figure 1

correspond to figure 1, panel E – top panel

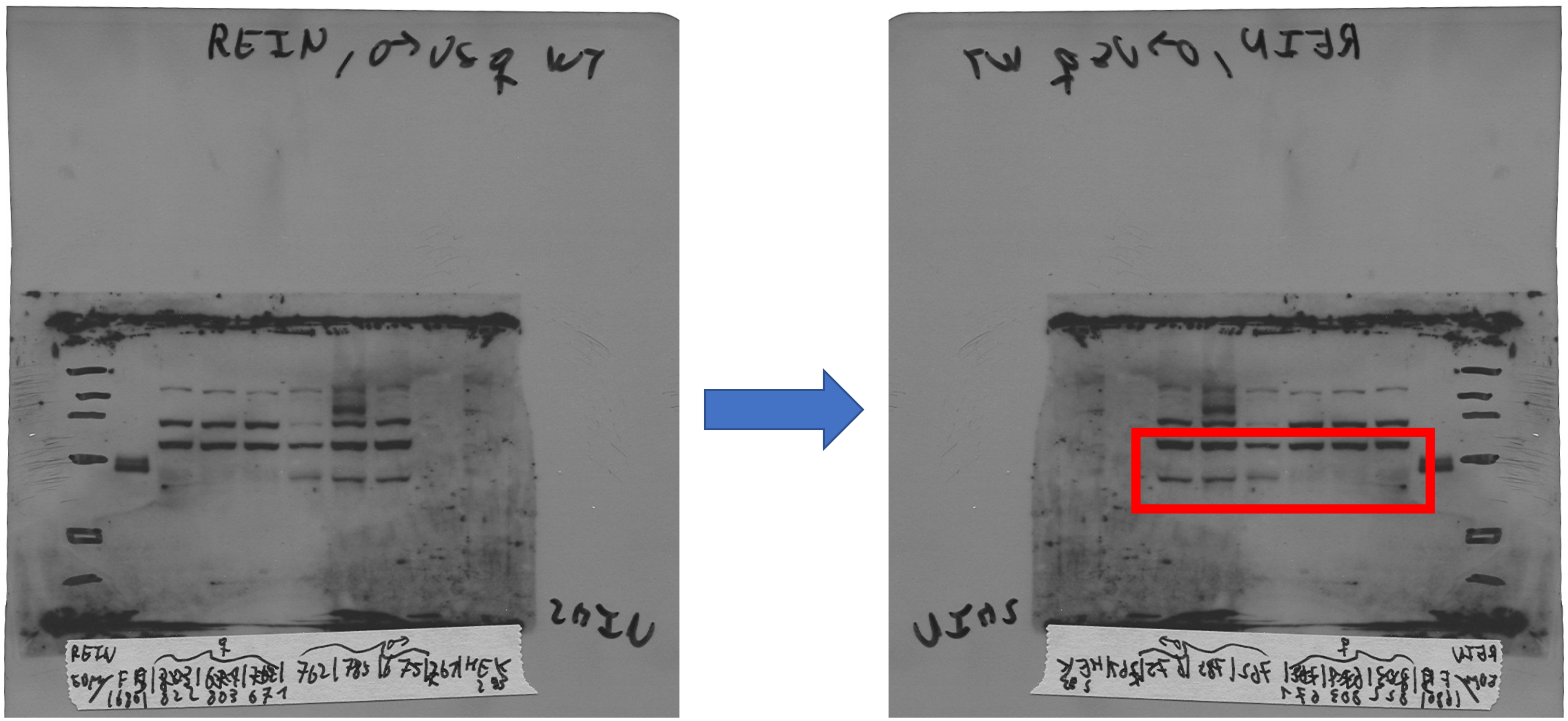

SUPPLEMENTARY FIGURE S1: Full western blots of Figure 1

correspond to figure 1, panel E – lower panel

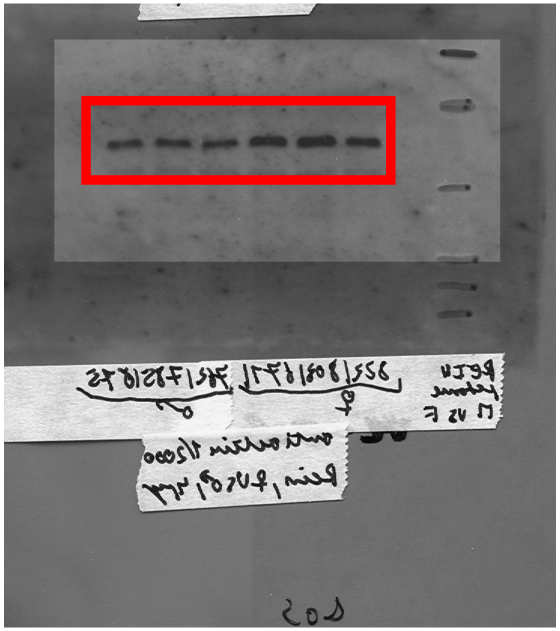

SUPPLEMENTARY FIGURE S2: Full western blots of Figure 3

correspond to figure 3, panel A

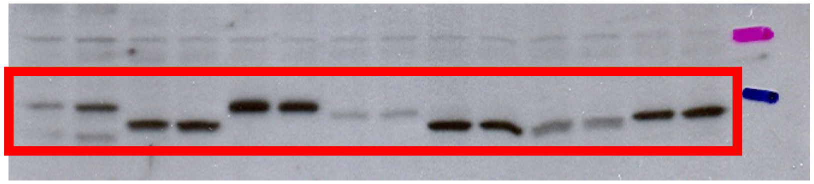

Supplement: Supplementary file 1 [file biology-11-00403-s001.zip › biology-1490217-supplementary.pdf]
